# Attenuation of Thrombosis by Crude Rice (*Oryza sativa*) Bran Policosanol Extract:* Ex Vivo* Platelet Aggregation and Serum Levels of Arachidonic Acid Metabolites

**DOI:** 10.1155/2016/7343942

**Published:** 2016-10-05

**Authors:** Wai-Teng Wong, Maznah Ismail, Eusni Rahayu Mohd Tohit, Rasedee Abdullah, Yi-Da Zhang

**Affiliations:** ^1^Laboratory of Molecular Biomedicine, Institute of Bioscience, Universiti Putra Malaysia, 43400 Serdang, Selangor, Malaysia; ^2^Department of Nutrition and Dietetics, Faculty of Medicine and Health Sciences, Universiti Putra Malaysia, 43400 Serdang, Selangor, Malaysia; ^3^Department of Pathology, Faculty of Medicine and Health Sciences, Universiti Putra Malaysia, 43400 Serdang, Selangor, Malaysia; ^4^Department of Veterinary Laboratory Diagnostics, Faculty of Veterinary Medicine, Universiti Putra Malaysia, 43400 Serdang, Selangor, Malaysia; ^5^Cardiology Department, Affiliated Hospital of Chengde Medical University, Chengde, Hebei 067000, China

## Abstract

*Background*. Vascular occlusion or thrombosis was often attributed to uncontrolled platelet activation. Influence of sugarcane policosanol extract on platelet was reported but little was known of rice bran policosanol, particularly its mechanisms of actions on platelet activities.* Objective*. Antiplatelet mechanisms of rice bran policosanol extract (RBE) were studied using hyperlipidemic Sprague Dawley rats.* Ex vivo* platelet aggregation, platelet count (PC), bleeding time (BT), and coagulation time were assayed. Serum eicosanoids and other aggregation-related metabolites levels were quantified.* Design*. Rats were divided into 6 groups for comparisons (vehicle control Tween 20/H_2_O, high dose policosanol 500 mg/kg, middle dose policosanol 250 mg/kg, low dose policosanol 100 mg/kg, and positive control aspirin 30 mg/kg).* Results*. Low dose 100 mg/kg of RBE inhibited aggregation by 42.32 ± 4.31% and this was comparable with the effect of 30 mg/kg aspirin, 43.91 ± 5.27%. Results showed that there were no significant differences in PC, BT, and coagulation time among various groups after RBE treatment. Serum thromboxane A_2_ was attenuated while prostacyclin level increased upon RBE treatment.* Conclusions*. RBE reduced* ex vivo *ADP-induced platelet aggregation without giving adverse effects. No changes in full blood count suggested that rice bran policosanol did not disturb biological blood cell production and destruction yet it reduced aggregation through different mechanisms.

## 1. Introduction

Inflammation, atherosclerosis, and thrombosis are all viewed as cardiovascular manifestations demonstrated by a cluster of risk factors. The prevalence and fatality rate of cardiovascular diseases (CVDs) worldwide, for instance, stroke and coronary heart disease, urged the progression in medical research [1]. CVD is a disorder caused by a sequence of biological events associated with oxidative stress, low density lipoprotein (LDL) deposition, tissue injury, foam cell formation, plaque formation, and so forth. Hence, compounds with antioxidant, anti-inflammatory, hypolipidemia, and/or antithrombotic properties are valuable [2].

In biological perspective, free flow of blood is of utmost importance. Platelets, smallest type of blood cell, play essential role in regulating smooth blood circulation, vascular integrity, and hemostasis [3]. However, uncontrollable hyperactivity of platelets beyond normal biological responses in the presence of platelet activators, for instance, free radicals, arachidonic acid, adenosine diphosphate, collagen, diabetic, and hyperlipidemic-related risk factors, significantly contributes to platelet dysfunctions and causes a series of atherothrombotic diseases [4].

Antiplatelet agents (APA) including aspirin, clopidogrel, and cilostazol are often used in prescription and remained as mainstay medication in vascular thrombotic diseases. However, the various side effects of such medications, for example, internal bleeding, rash, drowsiness, and bruising, impede their wide use in clinical aspects [5]. Therefore, there is an urgent need in finding antiplatelet agents from natural origin (plant bioactive compounds) to establish a novel, safer, and effective approach.

Natural antiplatelet agents (APA) that modulated platelet functions attained great attention from the public in primary and secondary prevention of cardiovascular diseases (CVD). Policosanol, a generic term for a mixture of long chain aliphatic alcohols (C20–C30), is usually extracted from animal and plant waxes, for example, sugarcane, wheat varieties, cereal, yam, and beeswax [6]. Policosanol is long-known for its physiological benefits such as cholesterol lowering property [7] and improvement of muscle endurance [8] as reviewed by Janikula [9]. In this study, rice bran policosanol extract, with its own unique fatty alcohols fingerprint, was studied* ex vivo* and* in vivo* for its effects on platelet functions using high fat diet-induced hyperlipidemic rat model.

## 2. Materials and Methods

### 2.1. Materials

ADP was purchased from Chronolog Incorporation (Havertown, PA, USA). Aspirin was a generous gift from Mr. Choy. All the reagents and solvents used in extraction and analysis were analytical grade purchased from Merck (Darmstadt, Germany). Rice bran was obtained from Bernas milling factory in Kuala Selangor, Selangor, Malaysia. All the consumables used in animal studies were supplied by Takrif Bistari Sdn Bhd (Selangor, Malaysia). Mazola corn oil (Unilever Malaysia), Nespray fortified milk powder (Nestle Malaysia), and Specialty feeds (TN, USA) were used in high fat diet preparation.

### 2.2. Methods

#### 2.2.1. Rice Bran Policosanol Extraction

Policosanol was extracted using solid-liquid extraction according to the described procedures minor modifications [10]. Rice bran sample with size of 4 mm was stabilized by heat treatment using an automated microwave oven (microwave conditions: 2450 MHz, 550 W, 110°C, 200 seconds). Briefly, 10 g of rice bran was placed in conical flask with 150 mL of hexane and methanol (20 : 1 v/v mixture). Extraction was performed by sonication technology (50 Hz, 350 W, 50°C, 3 hours) using Power Sonic 505 ultrasonicator (Hwashin Technology Co., Seoul, Korea). The rice bran residues were removed from the solvent extract by centrifuging at 4000 rpm for 10 minutes. The solvent was removed from the extract using a rotary evaporator (Buchi, Switzerland) under a vacuum at 40°C until greenish-yellow extract was seen.

#### 2.2.2. Preparation of High Fat Diet (HFD)

HFD was formulated following the recipe by Levin et al. [11] and prepared in accordance to Imam and Ismail [12]. Every kg of HFD was prepared from a mixture of 500 g of normal rat pellet, 240 g of corn oil, 200 g of full-cream milk powder, 60 g of sugar, and 50 g of starch. The HFD was cut and dried overnight in an incubator at 60°C.

#### 2.2.3. Animal Handling and Treatment

Experiments were conducted in accordance with the guidelines for animal use and ethical approval was obtained from institutional animal care and use committee (IACUC), Faculty of Medicine and Health Sciences, Universiti Putra Malaysia, Malaysia. The animals (36 adult male Sprague-Dawley rats) with body weight between 100 and 150 g were randomly divided into six experimental groups. All the rats were acclimatized for 7 days* ad libitum* with standard rodent chow and free access of clean water. These animals were kept in a temperature-controlled room (22 ± 2°C) with a 12 h light-dark cycle. The study was carried out for 3 months. Starting from second month, treatment was given once a day accordingly through oral gavage continuously for one month. Animal food remained the same throughout the whole experimental period: (1) a normal group receiving rodent chow and treated with vehicle (Tween 20/H_2_O); (2) a negative control group receiving HFD and treated with vehicle (Tween 20/H_2_O); (3) a positive control group receiving HFD and treated with aspirin (30 mg/kg); (4) a group receiving HFD and treated with 500 mg/kg policosanol; (5) a group receiving HFD and treated with 250 mg/kg policosanol; (6) a group receiving HFD and treated with 100 mg/kg policosanol. Suspensions were prepared daily 1 h before administration [13].

#### 2.2.4. *Ex Vivo* Platelet Aggregation

Platelet aggregation study was done using ADP as agonist according to Vaiyapuri and Gibbins [14] with minor modifications. Rat whole blood was collected into a vacutainer tube containing 3.2% sodium citrate (9 : 1, v/v) and mixed thoroughly by inverting the tubes for several times. The blood was centrifuged at 100 ×g for 20 minutes to obtain platelet rich plasma (PRP). 50 ng/mL of prostacyclin was added to prevent platelet activation. PRP was centrifuged again at 240 ×g for 10 minutes to sediment platelet pellet. The platelet pellet was resuspended in Tyrode-HEPES buffer and adjusted to a cell density of 10^8^ cells/mL. BioTeK Synergy H1 Hybrid Reader (BioTek Instruments Inc., Winooski, VT, USA) was used as analytical tool. 100 *μ*L of platelet cells was loaded into each well and 1 *μ*L of ADP was added in. The optical densities at 405 nm were read for 20 minutes. Reading was taken at a fixed time interval of 1 minute. The plate was shaken in double orbital mode at a frequency of 282 cpm, 3 mm.

#### 2.2.5. Full Blood Count (RBC, MCV, RDW, HCT, PLT, MPV, HGB, MCH, and MCHC)

Full blood count was done using hematology analyzer (Medonic CA530). Fresh rat blood was withdrawn into EDTA anticoagulated blood tubes and analyzed within 4 hours. Baseline and final readings were taken before and after treatment for comparison.

#### 2.2.6. Tail Bleeding Time Assay

Rat tail bleeding time was measured according to the described procedures with minor modifications [15]. Tail of anesthetized rat was warmed to 37°C. Bleeding time was determined by cutting 2 mm of the tail tip with a blade and blood was blotted onto a filter paper every 30 s until no bloodstain was seen. The period between amputation and the stop of bleeding was the bleeding time (min). Baseline and final readings were taken before and after treatment.

#### 2.2.7. Coagulation Time

Extrinsic and intrinsic pathways of coagulation were analyzed by measuring prothrombin time (PT) and activated partial thromboplastin time (aPTT). PT and aPTT were analyzed followed the standard protocol of Synergy Start 4 hemostasis analyzer (Diagnostica Stago Inc.).

#### 2.2.8. Quantification of Serum Eicosanoids, von Willebrand Factor (vWF), and Soluble p-Selectin

Stable platelet metabolites, thromboxane B_2_ and 6-keto PGF1*α*, were measured due to short half-life of thromboxane A_2_ and prostacyclin. Serum vWF and soluble p-selectin levels were quantified as an indication of* in vivo* platelet activation. Analysis was all done followed the kit's protocol using serum samples. Absorbance readings were obtained using BioTeK Synergy H1 Hybrid Reader (BioTek Instruments Inc., Winooski, VT, USA) at a wavelength of 450 nm for all kits.

## 3. Statistical Analysis

The data were analyzed using minitab 16 (Minitab Inc., State College, Pennsylvania, United States) by one-way analysis of variance (ANOVA) and the values were presented as means and standard deviation (SD). The significance of the differences between comparisons was determined by Tukey's range test. The significant difference was taken to be a value of *p* < 0.05 at a 95% confidence interval.

## 4. Results and Discussion

**Figure 1:**
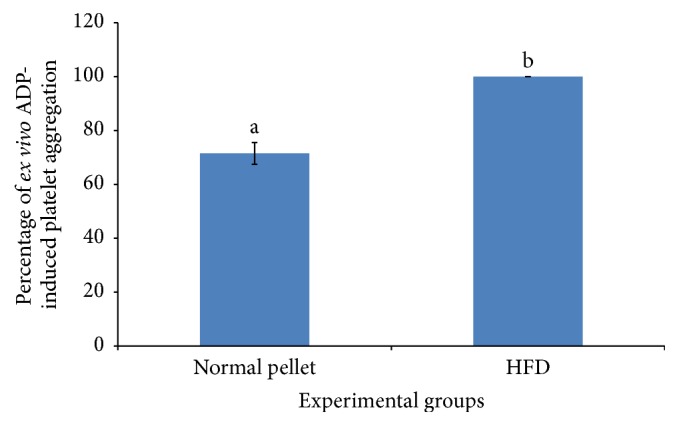
Percentage of* ex vivo* ADP-platelet aggregation (baseline: day 0 of extract treatment after two months of high fat induction) of HFD-fed rats and normal pellet-fed rats. Means labeled with different letters showed significant difference at *p* < 0.05.

Platelet, despite being the smallest type of blood cell, exerts enormous influence on physiology. Disturbance of platelet functions is attributed to a series of ischemic events [16]. Based on the presented value in Figure 1, two months of HFD successfully induced a significant higher degree of ADP-induced* ex vivo* platelet aggregation, which was about 30% increment in comparison to rat group consumed normal pellet. This was in agreement with Aoki et al. [17] that HFD increased platelet reactivity and thereby supporting the use of this animal model in screening antiplatelet agent. It has been reasonably postulated that people consuming oily food regularly were prone to obesity and cardiovascular diseases.

**Figure 2:**
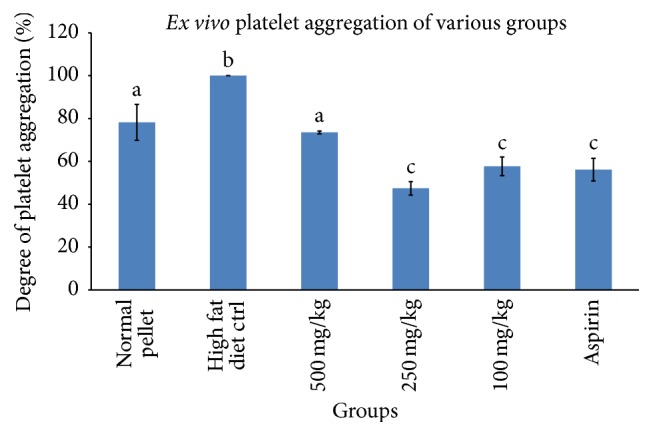
Degree of* ex vivo* ADP-induced platelet aggregation after a month of RBE and aspirin treatment (day 30 of treatment). Aspirin (30 mg/kg) was used as positive control and means that shared the same letter were not significantly different at *p* < 0.05 (*n* = 6).

The inhibitory action of rice bran policosanol extract (RBE) on* ex vivo* platelet aggregation was shown in Figure 2. RBE (100, 250, and 500 mg/kg) administered orally significantly inhibited ADP-induced platelet aggregation. Surprisingly, from the data obtained, dose 250 mg/kg inhibited platelet aggregation to the highest extent (~53%) and this can be explained by hormesis effect. Aspirin (30 mg/kg), a widely used APA, was used as positive control and it successfully inhibited platelet aggregation towards ADP by ~44%. The inhibitory power of 30 mg/kg aspirin was compared to 100 mg/kg of RBE.

**Figure 3:**
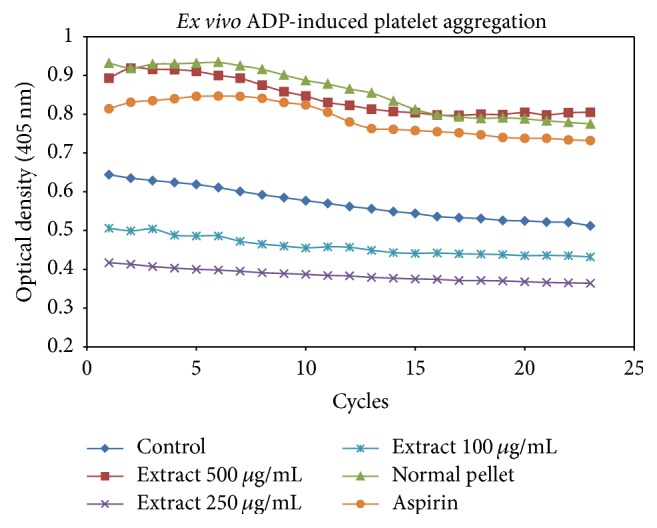
*Ex vivo* ADP-induced platelet aggregation patterns obtained in double orbital shaking mode using microplate reader at wavelength of 450 nm. Optical densities of 100 *μ*L of platelets suspensions from each group (*n* = 6) were studied for 23 cycles with a fixed time interval of one minute.

Figure 3 showed the aggregation patterns of platelet suspension over time stimulated with ADP and reduction in optical densities indicated that platelets aggregated upon agonist addition. However, RBE attenuated platelet aggregation and thus minute absorbance difference was found across the time. These data suggested that RBE was absorbed into the blood circulation and was a potent APA as it inhibited aggregation for certain extent as compared to HFD control group. Gastrointestinal metabolization and degree of absorption were the main determinants in controlling the concentration of bioactive compounds in blood and its efficacy as well [18].

**Figure 4:**
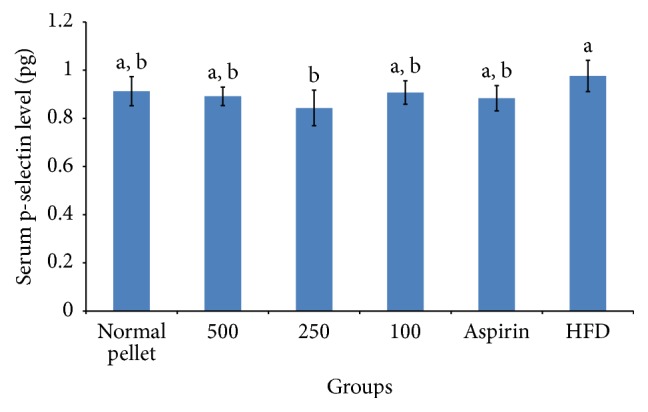
Serum level of soluble p-selectin after 30 days of RBE extract or aspirin treatment on experimental rats. Means which shared the same letter were not significantly different at *p* < 0.05.

**Figure 5:**
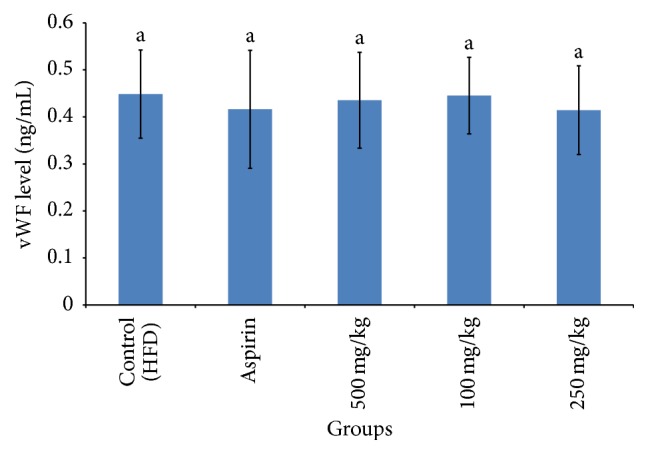
Serum level of soluble von Willebrand factor (vWF) after 30 days of RBE extract or aspirin treatment on experimental rats. Means which shared the same letter were not significantly different at *p* < 0.05.

Platelet activation was always accompanied with p-selectin expression. Measurement of soluble p-selectin is a form of important marker in determining the risk of thrombotic disorders as activated platelet was shown to shed their membrane bound p-selectin into blood plasma [19]. Less p-selectin exposure, or in an easy term less platelet activation, was important in reducing atherosclerotic lesions as p-selectin was a strong adhesive molecule in forming stable platelet aggregates and vital in platelet-monocyte interaction [20, 21]. From Figure 4, 250 mg/kg oral ingestion of RBE was shown to reduce soluble p-selectin detected in serum significantly by 14%. Influence of other activation factors, for example, centrifugal speed and temperature shock during blood serum collection, can be ignored as high threshold was needed to activate alpha granule and expose p-selectin, thus supporting the validity of the present study [22]. The soluble p-selectin detected was assumed to be originated from activated platelets instead of activated endothelial cells based on the presented value shown in Figure 5 and this was concurred with the study done by Perumal et al. [23]. There was no significant difference in von Willebrand factor (vWF) level detected in the blood serum between various groups at *p* < 0.05. This result highlighted that there was no endothelial activation and again proved our hypothesis on antiplatelet function of RBE.

As shown in Table 1, RBE treatments did not modify platelet number (10^9^/L) in comparison with HFD control group. This showed that the attenuation of platelet aggregation was not associated with platelet production or destruction* in vivo* upon RBE treatment. In addition to that, there were no statistical differences on various blood parameters between RBE-treated groups and control group. These suggested that RBE was safe for consumption (not toxic to cells).

Bleeding time, the time taken for bleeding to stop, was an important test in platelet function assessment [24]. Based on the data depicted in Table 2, RBE did not significantly prolong bleeding time as compared to control group at *p* < 0.05. This result was correlated with other literatures stating that antiplatelet compounds were not often associated with prolongation of bleeding time [18, 25, 26]. On the other hand, RBE and aspirin did not significantly affect PT and aPTT, and this showed that RBE potentiated antiplatelet effect without influencing coagulation system. These notable findings supported the consumption of brown rice or rice bran-derived products as therapeutic approach without giving adverse side effects.

**Figure 6:**
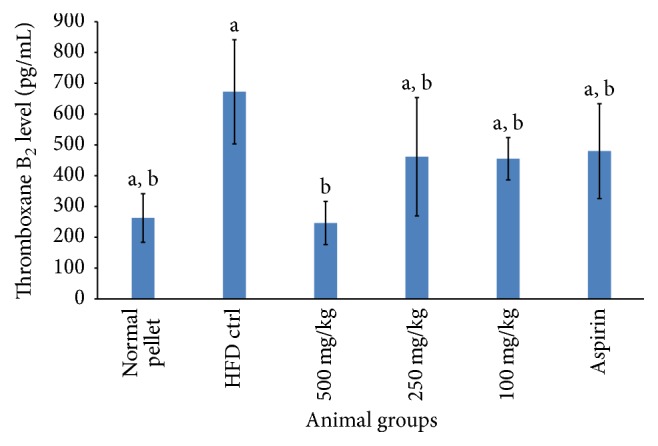
Serum level of thromboxane B_2_ after 30 days of aspirin/RBE treatment, *n* = 6. Means which shared the same letter were not significantly different at *p* < 0.05.

**Figure 7:**
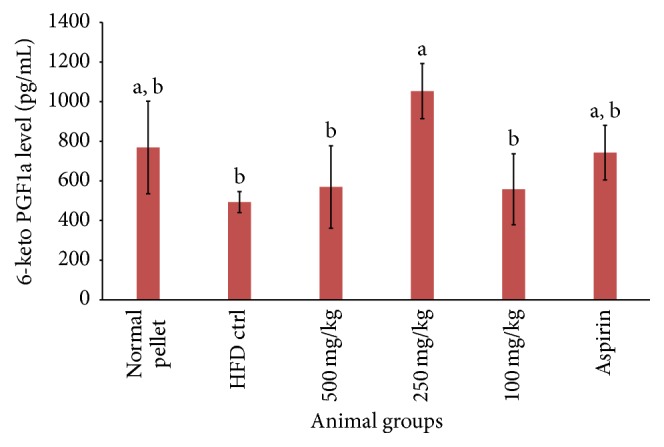
Serum level stable metabolite of prostaglandin I_2_ (6-keto PGF1*α*) after 30 days of aspirin/extract treatment, *n* = 6. Means which shared the same letter were not significantly different at *p* < 0.05.

Practically, platelets were activated by numerous physiological factors through a multitude of mechanism, for instance, interaction with platelet receptors or glycoproteins, modulation of intracellular platelet messengers, and regulation of platelet signaling products. Despite different pathway of platelet activation, platelet responded in the same series initiated with shape change, secretion, liberation including prostaglandins, or lipooxygenase products, followed by aggregation [27]. RBE was a crude extract containing a mixture of bioactive compounds and policosanol was understood to be the leading compound responsible in exerting antiplatelet function. However, existence of other bioconstituents, for example, polyphenolic compounds or tocopherols, might work individually or synergistically in giving antiaggregant activity [28, 29]. The actual mechanism by which RBE implicated in antiplatelet function was yet to be known. Figures 6 and 7 showed that RBE significantly reduced serum thromboxane B_2_ and meanwhile increased prostacyclin level. These results were concurred with other studies [30–32]. The results suggested that RBE exerted its antiplatelet function through mechanisms different from aspirin (ASA). Reduced TXA_2_/PGI_2_ ratio as compared to control inhibited pathophysiology ischemic events is associated with platelet activation [13].

From the result obtained, we can boldly hypothesize that RBE might serve as antagonists of receptor GPIIb/IIIa (fibrinogen receptor), P_2_Y_1_ and P_2_Y_12_, thus preventing the ADP-induced* ex vivo* platelet aggregation and thromboxane B_2_ liberation. It was believed that both inside-out signaling through ADP receptors and outside-in signaling through fibrinogen receptor were necessary to activate phospholipase A_2_ activity and result in thromboxane A_2_ generation [33]. In addition to that, antagonism of receptor P_2_Y_12_ increased intracellular cAMP level and further reduced platelet aggregation by regulating a series of enzyme activities [34].

**Figure 8:**
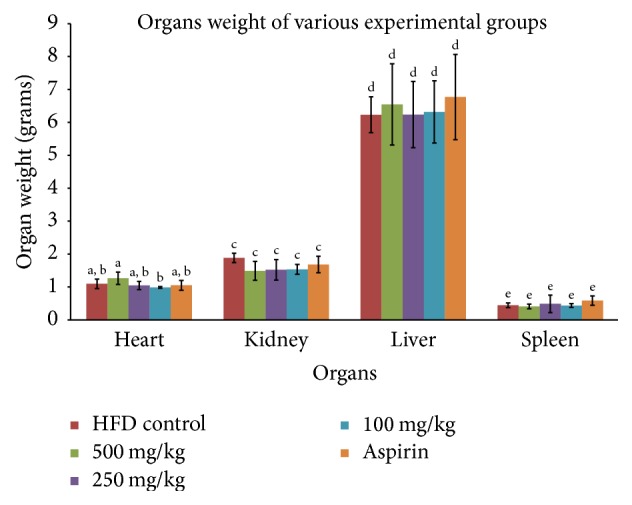
Organs weight (heart, liver, spleen, and kidney) of sacrificed rats from various experimental groups. The presented values were mean ± standard deviation and means denoted with same letters were not significantly different at *p* < 0.05 (*n* = 6).

**Figure 9:**
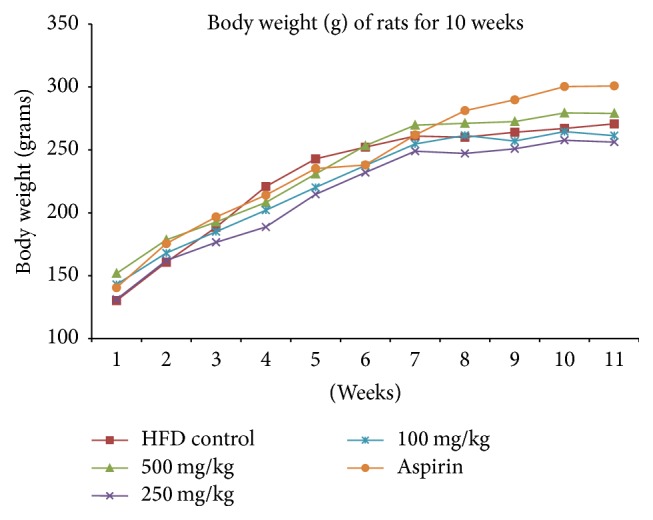
Increasing trend of the body weight (in grams) of rats from various experimental groups (negative control group, policosanol-treated groups, and aspirin-treated group), *n* = 6.

Additionally, antioxidant ability of RBE was a potential factor in reducing platelet aggregation. Inhibition power of RBE on endogenous and exogenous free radicals from attacking platelets must not be discarded [35]. On the other hand, long believed hypocholesterolemic action (reduction in LDL-C, total cholesterol, and triglycerides) and endothelial protecting action of policosanol made RBE a potential agent to reduce* in vivo* platelet activation [36–38]. RBE showed no toxicity to experimental animals as shown in Figures 8 and 9. Normal increment in rat body weight along the study period suggested the safety use of RBE in human. Holistically, the present study suggested the feasibility of RBE as antiplatelet agent as it selectively inhibited platelet-dependent mechanisms without disturbing the normal physiology.

## 5. Conclusion

The present study demonstrated that rice bran policosanol extract (RBE) potentiates antiplatelet effect using* ex vivo* and* in vivo* models. Antiplatelet effect of RBE was evident after 30 days of RBE oral administration. RBE successfully reduced serum thromboxane A_2_ production but increased prostacyclin level. Notably, significant platelet aggregation inhibition did not accompany the prolongation of bleeding time and coagulation time and thus overwhelmed other common APA. This research provided great insights into developing a novel approach which fundamentally could reduce the adverse side effects while retaining its effectiveness.

## Figures and Tables

**Table 1:** Full blood count analysis using hematology analyzer (baseline: day 0 of treatment; final reading: day 30 of treatment).

Groups	RBC (10^12^/L)	MCV	RDW	HCT	PLT (10^9^/L)	MPV (fl)	HGB (g/L)	MCH (pg)	MCHC (g/L)
		(fl)	(%)	(%)					
NP (before treatment)	8.005 ± 0.04^a^	53.95 ± 0.35^b^	10.7 ± 2.12^c^	40.85 ± 3.75^d^	712 ± 97.58^e^	6.7 ± 0.14^f^	148.5 ± 9.19^g^	19.75 ± 0.64^h^	367 ± 14.14^i^
HFD (before treatment)	7.49 ± 0.68^a^	48.58 ± 1.36^b^	14.35 ± 1.32^c^	36.21 ± 3.23^d^	844.25 ± 157.46^e^	6.93 ± 0.12^f^	133.78 ± 10.45^g^	17.91 ± 0.78^h^	366.3 ± 6.70^i^
NP (after treatment)	7.21 ± 1.01^a^	52.97 ± 1.89^b^	13.83 ± 2.46^c^	38.1 ± 4.42^d^	730 ± 97.58^e^	7.27 ± 0.45^f^	138.67 ± 20.50^g^	19.27 ± 0.72^h^	363.67 ± 13.65^i^
HFD-NC (after treatment)	7.8 ± 0.71^a^	49.47 ± 1.29^b^	13.53 ± 0.67^c^	38.53 ± 2.51^d^	1155.33 ± 160.55^e^	6.77 ± 0.06^f^	143.33 ± 8.08^g^	18.37 ± 0.67^h^	372 ± 3.61^i^
500 mg/kg	7.66 ± 1.04^a^	47.27 ± 1.88^b^	15.53 ± 0.67^c^	36.3 ± 6.16^d^	756 ± 86.27^e^	7.53 ± 0.91^f^	131.67 ± 23.44^g^	17.13 ± 1.03^h^	362.33 ± 8.74^i^
250 mg/kg	8.29 ± 0.25^a^	47.37 ± 1.25^b^	14.87 ± 0.84^c^	39.3 ± 1.31^d^	961 ± 147.89^e^	7.13 ± 0.40^f^	143.33 ± 4.62^g^	17.27 ± 0.55^h^	365 ± 2^i^
100 mg/kg	7.86 ± 0.31^a^	48.23 ± 1.32^b^	15.23 ± 1.67^c^	37.9 ± 1.4^d^	772 ± 288.5^e^	7.13 ± 0.61^f^	138 ± 6.08^g^	17.57 ± 0.40^h^	364 ± 3.46^i^
Aspirin-PC	8.31 ± 0.23^a^	46.53 ± 0.81^b^	15.3 ± 1.42^c^	38.67 ± 0.64^d^	902 ± 106.07^e^	7 ± 0.7^f^	140.33 ± 3.51^g^	16.87 ± 0.23^h^	363 ± 3.61^i^

NC: negative control; PC: positive control; NP: rats which consume normal pellet; HFD: rats which consume high fat diet throughout 3 months study period, *n* = 6. The presented value was mean ± standard deviation. Means that shared the same letter were not significantly different at *p* < 0.05.

**Table 2:** Tail bleeding time (BT), activated partial prothrombin time (aPTT), and prothrombin time (PT) in secs of various experimental groups after oral administration of RBE or aspirin for one month, *n* = 6.

Groups	BT (secs)	aPTT (secs)	PT (secs)
HFD-negative control	484 ± 45.36^a^	19.7 ± 1.8^b^	19.8 ± 0.8^c^
500 mg/kg	573.6 ± 39.11^a,b^	16.8 ± 3.1^b^	17.7 ± 1.5^c^
250 mg/kg	540.8 ± 93.59^a,b^	17.5 ± 1.8^b^	18.8 ± 0.2^c^
100 mg/kg	521.2 ± 97.16^a,b^	16.3 ± 0.3^b^	17.7 ± 1.6^c^
Aspirin-PC	597 ± 96.77^b^	15.1 ± 0.1^b^	18.4 ± 0.8^c^

The presented value was mean ± standard deviation. Means that shared the same letter were not significantly different at *p* < 0.05.
